# Attention sports fans! The far-reaching contributions of bud sport mutants to horticulture and plant biology

**DOI:** 10.1038/s41438-018-0062-x

**Published:** 2018-07-15

**Authors:** Toshi M. Foster, Maria José Aranzana

**Affiliations:** 1 The New Zealand Institute for Plant and Food Research Limited, Private Bag 11600, Palmerston North, 4474 New Zealand; 2 IRTA (Institut de Recerca i Tecnologia Agroalimentàries), Barcelona, Spain; 3 Centre for Research in Agricultural Genomics (CRAG) CSIC-IRTA-UAB-UB, Campus UAB, Bellaterra, Barcelona, Spain

## Abstract

A bud sport is a lateral shoot, inflorescence or single flower/fruit with a visibly different phenotype from the rest of the plant. The new phenotype is often caused by a stable somatic mutation in a single cell that is passed on to its clonal descendants and eventually populates part or all of a meristem. In many cases, a bud sport can be vegetatively propagated, thereby preserving the novel phenotype without sexual reproduction. Bud sports provide new characteristics while retaining the desirable qualities of the parent plant, which is why many bud sports have been developed into popular cultivars. We present an overview of the history of bud sports, the causes and methods of detecting somaclonal variation, and the types of mutant phenotypes that have arisen spontaneously. We focus on examples where the molecular or cytological changes causing the phenotype have been identified. Analysis of these sports has provided valuable insight into developmental processes, gene function and regulation, and in some cases has revealed new information about layer-specific roles of some genes. Examination of the molecular changes causing a phenotype and in some cases reversion back to the original state has contributed to our understanding of the mechanisms that drive genomic evolution.

## Plant biology: The value of good sports

Careful analysis of unusual shoots on parent plants provides valuable insights into mechanisms driving genomic evolution. Sometimes, plants produce shoots, single flowers or fruit that are visibly and genetically different from the rest of the plant. These ‘bud sports’ are caused by stable mutations and usually retain the positive characteristics of the parent plant. They are useful for cultivating novel varieties, particularly in species with low genetic variability. Toshi Foster at the New Zealand Institute for Plant and Food Research in Palmerston North, New Zealand, and Maria Jose Aranzana at the Center for Research in Agricultural Genomics in Barcelona, Spain, reviewed current knowledge of bud sports and their genetic origins. Analyses of buds using next-generation sequencing is providing insights into the function and regulation of genes and mutations, with applications in agriculture and plant biology.

## Introduction

The use of traditional breeding to improve the quality of perennial fruit, nut, or ornamental plants is hindered by several factors. Many perennial species have a long juvenile period and generation time, some are self-incompatible, and most are highly heterozygous, which means valuable qualities may be lost through sexual reproduction. For these reasons, many woody perennials are vegetatively propagated by cutting, grafting, and budding, which can preserve desirable genotypes over long periods of time. Indeed, there are examples of wine grapes that have been clonally propagated for centuries^1,2^.

Occasionally, a lateral shoot, inflorescence or single flower/fruit is discovered with a visibly different phenotype from the rest of the plant. These are called bud sports and are often caused by a stable somatic mutation in a single cell that is passed on to its clonal descendants and eventually populates part or all of a meristem, enabling vegetative propagation of the new mutant. Although these are relatively rare events, sport mutations often provide valuable new characteristics while retaining the desirable qualities of the parent plant. Therefore, somatic mutation represents a mechanism to generate new genetic variability which is especially important for species with low levels of variation. Many economically important perennial cultivars are bud sports^3,4^. By 1936 there were at least 1664 known fruit tree bud sports, representing 32% of the plant patents issued by the U.S. Patent Office at that time^5^. More recently, Okie^6^ reported that more than 170 commercialized cultivars of peach and nectarine are derived from bud sport mutations.

The identification of bud sports relies on astute observation by the grower, breeder, or gardener. While these have likely been observed since humans have been cultivating plants, the earliest report of a bud sport was published by the botanist Gaspard Bauhin in 1598 and describes the unusual leaf phenotype of a *Chelidonium majus* (celandine) plant found in a herb garden^7^. In 1644, gardener Pietro Nati noticed a shoot bearing unusual fruit (the “Bizarria” orange) growing from the graft junction of two types of citrus^8^. Charles Darwin was fascinated by “sporting plants” and published numerous reports of spontaneous mutants^9–12^. In his famous book, *The Variation of Plants and Animals Under Domestication*^11^, Darwin noted that “Many cases have been recorded of a whole plant, or a single branch, or bud suddenly producing flowers different from the proper type in colour, form, size, doubleness, or other character. Half the flower, or a smaller segment sometimes changes colour”. In some cases, the molecular or cytological change(s) causing the new phenotype have been identified, which has contributed to our knowledge of the mechanisms that lead to the formation of sports and provided novel information about gene function and regulation. This review will highlight some of these examples.

## Meristems make the plant

**Fig. 1:**
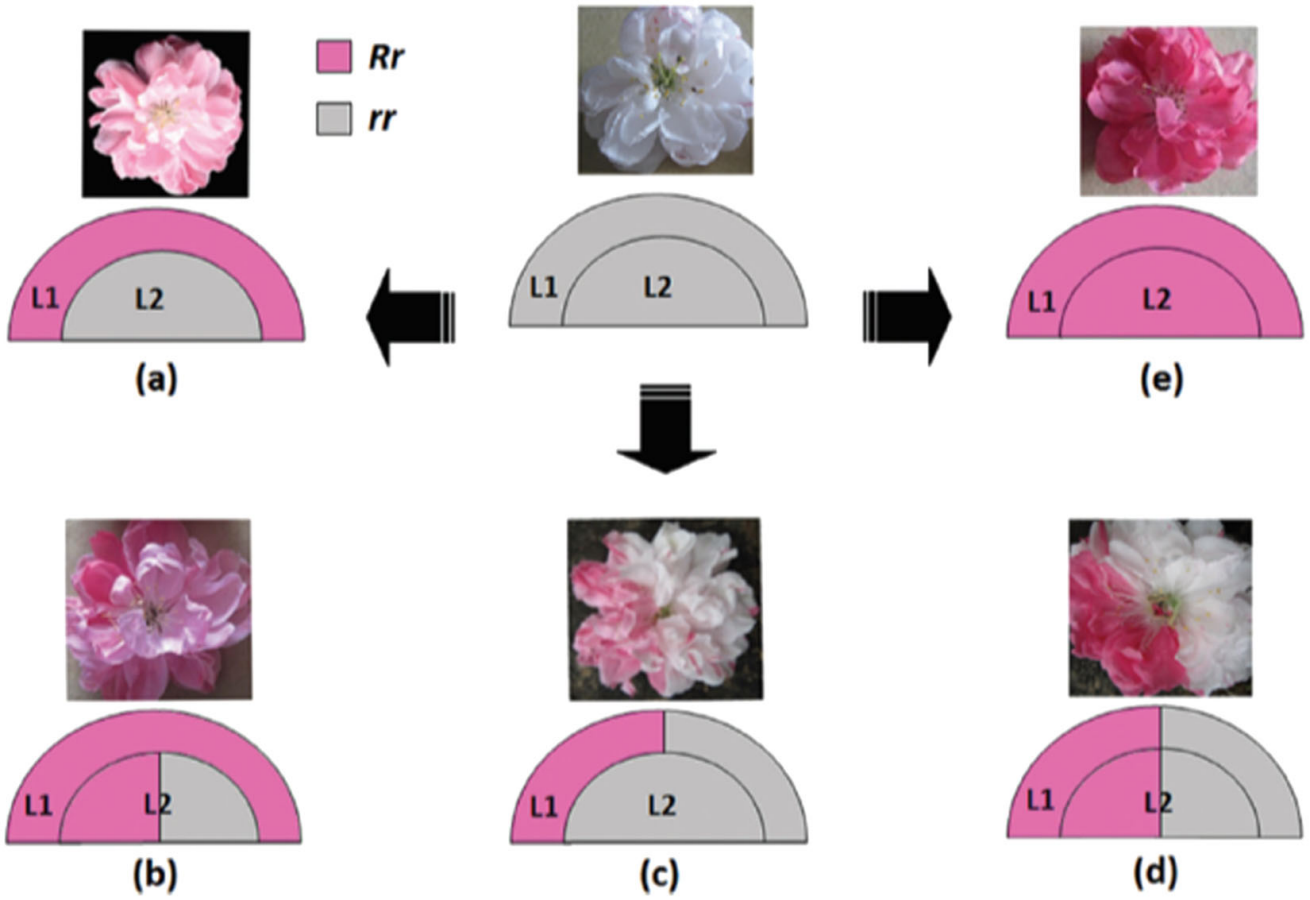
A proposed model for the variegated phenotype in flower colouration in peach cv. HBH. L1 and L2 indicate different layers of floral meristems, and *R* and *r* represent functional and non-functional alleles of the *RIANT* gene, respectively. White flower carrying two non-functional alleles of the *RIANT* gene (*rr*). **a** Pink flower derived from a periclinal chimera. **b** Pink flower with red somatic sectors derived from a mericlinal chimera. **c** White flower with pink somatic sectors derived from a mericlinal chimera. **d** White flower with red somatic sectors derived from a sectorial chimera. **e** Red flowering carrying one functional and one non-functional allele of the *RIANT* gene. *Source*: Cheng et al.^61^

Most of the above-ground parts of the plant are produced by clusters of rapidly dividing cells in the apical and axillary meristems. Angiosperm meristems are organized into one or more outer layers, the tunica, and an inner layer or corpus which reflect stereotypical patterns of cell division gleaned from histological analysis and cell lineage studies^13^. Cells in the tunica tend to divide anticlinally (new cell walls formed perpendicular to the surface) such that they form one or more clonally distinct layers (Fig. 1). The number of tunica layers ranges from one to five with most species having two, the L1 and L2. The L1 gives rise to the epidermis, the L2 generates sub-epidermal layers and the germline. The corpus or L3 divides in all planes and gives rise to cells that become the core of lateral organs and the cortex of the stem.

Superimposed over the tunica/corpus organization are distinct zones that are defined by histological properties and cell division rates. At the very apex of each layer, there are a very small number of cells that are larger and stain less densely than surrounding cells^13^. These initial cells divide slowly and act as a reservoir of stem cells that replenish the supply of rapidly dividing cells on the flanks and central zone of the meristem that eventually become incorporated into lateral organs and the stem^13^.

The analysis of chimeric plants comprised of cells with distinct genotypes and phenotypes has provided valuable insight into meristem organization^14^. For example, a somatic mutation occurring in a cell located close to the apex often results in a mericlinal sector, which is visible in a portion of an organ (Fig. 1b, c, d). Occasionally, a mutant cell will populate an entire layer, creating a periclinal or layer chimera that can remain stable for long periods of time (Fig. 1a–c). Adventitious shoots originating at graft junctions can produce graft chimeras, which are generally periclinal chimeras comprised of two distinct genotypes or even different species^15–18^. Two excellent reviews provide in-depth information about the use of both spontaneous and induced chimeras as a research tool^19^ and as a source of valuable new horticultural cultivars^20^. Mutant L2 periclinal chimeras enter the germline, and thereafter can be sexually propagated, leading to the mutation becoming fixed in subsequent generations. Periclinal chimeras can also become homogenized when mutant cells divide into adjacent layers and eventually displace wild-type cells.

It has been assumed that meristematic cells accumulate more mutations because they have high rates of mitosis. However, it has recently been shown that shoot apical meristem cells have a relatively low mutation rate even in large perennial plants^21,22^, which may explain the low rates of somatic variation found in long-lived trees^23^.

## Causes and identification of somaclonal variation

Mutations can be caused by changes to the DNA sequence or by epigenetic variations that modify DNA or histones and affect gene expression, but do not alter the sequence itself. DNA sequence errors may occur during replication, recombination, DNA damage repair during mitosis, and by transposable elements (TE). Additionally, epigenetic variations such as DNA methylation, histone modification, chromatin remodelling, and RNA silencing can cause stable changes in gene expression, generating sports.

The most frequent mutations caused by DNA polymerase errors are point mutations, tandem repeats, small insertions and deletions (indels), and base mismatches. Polymerase slippage is known to produce variability in simple sequence repeat (SSR) regions. For example, Aranzana and co-workers^24^ estimated a mutation rate of 1.1% of SSR alleles between peach sports. More recently, high-throughput next-generation sequencing (NGS) technologies have been used to estimate variability between bud mutants, providing more comprehensive information. Whole-genome sequences of “Fuji” apple and four bud mutant cultivars were compared to identify small polymorphisms, which revealed an average rate of eight single-nucleotide polymorphisms (SNPs) and 1.2 indels per Mb over the genome^25^. In grape, Carrier et al.^26^ compared whole-genome sequences of Pinot noir and three of its clones and found 1.6 SNPs and 5.1 indels per Mb.

Failures in the DNA replication process and damage caused by chemicals or radiation may produce DNA double-strand breaks (DSBs). Eukaryotic organisms have developed efficient mechanisms to repair DSBs through non-homologous end joining (NHEJ) and homologous recombination (HR) pathways. NHEJ is likely to occur more frequently than HR^27^ and does not require a homologous sequence to ligate the two DSB; it can result in small insertions or deletions of DNA at the break location and presumably contributes to microsatellite instability^28^. In contrast, the HR pathway requires an intact DNA molecule as template, such as the sister chromatid in a cell in S or G2 phases or the homologous chromosome. While recombination with the sister chromatid will not produce a mutation, recombination with the homologous chromosome may result in loss of heterozygosity or genome rearrangements^29,30^. Migliaro et al.^31^ characterized grape sports that were likely generated after independent DSBs and subsequent repair produced deletions ranging in size from a single bp to larger than Mbs along chromosome 2. More complex structural variations have been identified on three chromosomes of the grape sport Tempranillo blanco^32^, resembling a chromothripsis-like mechanism (clustered chromosomal rearrangements), which could have been generated by illegitimate re-joining of chromosome breaks in a unique event. Another example of structural variations that can alter gene expression and phenotype are copy number variants, which involve duplications or deletions of large segments of DNA^33,34^.

TEs contribute to genome plasticity and cause the majority of somatic variation in plants^25,26,35–37^. TEs can disrupt coding sequences, alter the expression of nearby genes and produce chromosome breakage leading to genome rearrangement and/or genome instability^38^. Retrotransposons are one class of TEs that are particularly disruptive because they replicate via reverse transcription of messenger RNAs and the duplicates integrate into other chromosomal locations. Related long terminal repeat (LTR) transposons are also sources of somatic variability as they can excise due to either homologous or illegitimate recombination between the terminal repeats, resulting in genomic loss or rearrangement. The activation and silencing of TEs is regulated by epigenetic mechanisms, therefore changes in such mechanisms may produce TE-driven somatic variability. Transcriptomic and epigenetic analyses have demonstrated variation in the transcription of genes associated with epigenetic mechanisms during bud dormancy release in fruit trees^39,40^, which may lead to the generation of sports. Stresses such as wounding, pruning, viral infection, and tissue culture are all known to induce the movement and/or activity of TEs^41,42^, as well as DNA damage^43^. McClintock^44^ first proposed that stress-induced activation of TEs could be a plant survival strategy to rapidly increase genotypic and phenotypic diversity in response to unfavourable conditions.

In most cases, bud sport mutations are genetically identical to their parent except for the new mutation; comparison of the parent and sport genotypes provides an opportunity to identify the molecular lesion responsible for the new phenotype. Several molecular and sequencing methods accompanied by bioinformatics pipelines have been developed to detect novel somatic changes. NGS technologies, especially those producing long, single-molecule reads using nanopore sequencing, have proven reliable in the detection of TEs that are transpositionally active in plants^45^, while new strategies are proposed to identify TEs active during plant development^46^. For a more precise estimation of somatic variability rates, such methods should consider the layer specificity of some mutations as in Marroni et al^47^.

Sports can arise from plants that are heterozygous for one or more loss-of-function mutation(s) that acquire independent new mutation(s) to the functional allele. Dominant gain-of-function mutations or a loss-of-function mutation to a haploinsufficient gene (one that requires two functional alleles to appear wild type) can produce a different phenotype with a single mutation event.

Lastly, not all bud sports are caused by genetic mutation. Hybridization between species and spontaneous changes in ploidy can introduce somatic variability leading to novel phenotypes^48^. Many sports originate as chimeras, comprised of cells from two distinct genotypes^14,20^. Most of the bud sports identified affect the fruit, probably because they are easy to observe, and many ornamental sports have altered floral or leaf phenotypes. In the sections that follow, we will group sports by their altered phenotype.

## Floral and inflorescence morphology and/or colour

Humans have appreciated the beauty, colour and aromatic scent of flowers since at least 12,000 years ago^49^. Many ornamental plant cultivars originated as bud sports that change the appearance of flowers or inflorescences. Wild roses have a single whorl of five petals, whereas most cultivated roses (*Rosa hybrida*) have many petals. Dubois^50^ and co-workers demonstrated that the rose orthologue of *AGAMOUS* (*RhAG*) is expressed in whorls 3 and 4 of wild roses, consistent with its role as a C-function gene that has a key role in specifying stamen and carpel identity^51^. In double-flower roses, *RhAG* expression is restricted to a much smaller domain in the centre of the floral meristem and whorls of stamens are converted to petals. Analysis of sports that revert back to five petals also show expanded *RhAG* expression into whorl 3^50^. The molecular lesion causing the misexpression of *RhAG* is unknown; identification of the mutation responsible may reveal information about the regulation of *RhAG* expression. The authors suggest that the boundary between A-function and C-function gene expression is very labile and might explain how double flower roses have arisen and been selected by humans more than twice.

Somatic grape mutants with altered flower and inflorescence development produce some of the most conspicuous phenotypes and illustrate the developmental plasticity of the tendril^52^. A somatic variant of Carignan has a reiterated reproductive meristem (RRM) phenotype, leading to large, highly branched fruit clusters and delayed anthesis. The tendrils of RRM mutants are also more indeterminate than wild type, displaying multiple branches or even conversion to a leafy shoot. The RRM phenotype is caused by insertion of a TE into the promoter of *VvTFL1A*, a close homologue of Arabidopsis *TERMINAL FLOWER1* (*TFL*)^35^. Genetic and genomic analyses demonstrate that the insertion of the transposon is associated with upregulation of *VvTFL1A*. This is consistent with previous studies showing that *TFL* genes control the length of developmental phases and maintain indeterminate inflorescence growth^53,54^.

Mutants of Gamay, Morrastel, and Pinot initiate extra whorls of sepals and petals and are collectively known as multiple perianth whorls^52^. Stamen and carpel development is abnormal in variants of Bouchalès and Mourvèdre, the latter being completely sterile. Although the molecular lesion(s) causing these phenotypes is unknown, the *MADS* box ABC genes would be prime candidates^55^.

More than half of commercial varieties of azalea (*Rhododendron simsii* hybrids) are colour sports^56^. Solid colour and variegated sports are likely L1 mutants because the new phenotype is not transmitted to progeny^57^. The variegated sports are likely to be transposon-mediated changes to pigment genes, although no direct evidence exists. The “picotee” phenotype is characterized by petals with a coloured centre and white margins. In broad-margined “picotee” mutants, the margin cells were found to be tetraploid and the coloured cells diploid, suggesting that positionally determined polyploidisation underlies this pattern. In general, coloured azalea sports were hypermethylated relative to their parent^57^. In carnation (*Dianthus caryophyllus*), the L1 layers showed very different patterns of methylation to L2 and L3 layers, and these patterns differed widely between sports and their parents^58^.

Ornamental flowering peach trees can produce white, pink, and red flowers on the same tree^59–61^. In some cases, this variability has been attributed to an unstable TE in the *W* locus^60^ or differential expression of transcription factors and genes in the anthocyanin biosynthesis pathway between red and white flowers^59^, but genetic lesions responsible have not been identified. In the “Hongbaihuatao” (HBH) cultivar, Cheng^61^ and co-workers identified a small indel in *RIANT*, a gene encoding an anthocyanin transporter required for pigment accumulation. White flowers are homozygous for a 2-bp insertion which introduces a frameshift mutation and a premature stop codon. Red and pink flowers are heterozygous at the *RIANT* locus, with one non-functional allele and a second allele with either a 1-bp insertion or a 2-bp deletion that restores gene function. Periclinal, mericlinal, and sectorial chimeras with or without *RIANT* function produce white, pink, and red flowers (Fig. 1). These mutations are not in a microsatellite region, but are in a CG-rich region, which could increase the rate of small indels^62^.

## Pollination, seedlessness, and fruit ripening

Self-incompatibility (SI) is a genetic mechanism that prevents inbreeding in some flowering plants. In most plants in which this SI mechanism operates, SI is controlled by a single, multi-allelic *S*-locus, which enables the pistil to reject pollen with the same S-allele^63^. Self-compatible sports have been identified in Japanese pear (*Pyrus serotina*)^64^ and almond (*Prunus delcis*)^65^. The pear sport is caused by genomic deletion of at least 4 kb that removes an S-RNase gene responsible for the SI reaction in the style. This mutation is only in the L1, providing evidence that the transmitting tissue in pear is L1-derived. In the self-compatible almond sport “Jeffries”, at least two mutations occurred, the deletion of one S haplotype and duplication of another, resulting in self-compatibility.

In most species, fruit and seed development are linked; however, there are examples where fruit development occurs in the absence of seed development. Other forms of SI occur after pollen germination and affect fertilization or embryo development, often leading to the development of a seedless fruit. Numerous seedless citrus sports have been identified, some of which have become popular cultivars such as satsuma mandarin (*Citrus reticulata*) and seedless navel orange. The Zigui shatian pummelo (*Citrus grandis* Osbeck) sport produces self-pollinated fruit, but the seeds are sterile because of defective post-zygotic development^66^. The seedless mandarin sports Ougan^67^, Wuzishatangju^68^, and Huami Wugegonggan^69^ are caused by pollen abortion, blocked fertilization, and pollen sterility and embryo abortion, respectively.

Bud sports that ripen earlier or later than their parents have been identified in numerous species. While many aspects of fruit ripening are directly controlled by the hormone ethylene^70,71^, different genotypes vary in their sensitivity to ethylene^72^. Fruit types with ripening traits predominantly regulated by ethylene are often termed “climacteric”, while those that are predominantly regulated by other factors are labelled “non-climacteric”. Genomic sequencing of bud sports of the Japanese plum “Santa Rosa” revealed copy number variation in genes associated with ethylene perception and signal transduction^73^. The non-climacteric and supressed-climacteric mutants had significantly fewer copies of 1-aminocyclopropane-1-carboxylic acid (ACC) oxidase (the enzyme that catalyses the final step in ethylene synthesis) and the ethylene receptor gene *ETHYLENE INSENSITIVE 1* (*ETR1*) relative to their climacteric parent. A number of frameshift mutations were also identified in genes involved with sugar transport and ethylene biosynthesis. Similarly, early-ripening “Beni Shogun” apples show increased expression of ethylene synthesis and signal transduction genes^74^. Late-ripening Tardivo mandarin sports are less sensitive to ethylene and have decreased expression of *ETR1* and *ETR2*^75,76^. Transcriptomic and proteomic analyses of several late-maturing bud sports of sweet orange have revealed differential expression of genes involved with abscisic acid (ABA), ethylene, and jasmonic acid (JA) synthesis and signal transduction, as well as sugar metabolism and carotenoid biosynthesis^77–79^.

## Altered fruit colour

Humans have been propagating grapevine (*Vitis vinifera* L.) for fresh fruit and wine making for over 10,000 years and from the second half of the 20th century clonal selection for wine grape breeding has been intensively used, so it is not surprising that many somatic variants have been identified that affect berry quality traits (mainly colour) and that these sports have been developed into cultivars. Grape berry colour is caused by the accumulation of anthocyanins in the berry skin (L1) and flesh (L2)^80^. Two tandemly repeated *Myb* regulatory genes, *VvMybA1* and *VvMybA2*, regulate red berry colour^81–83^. Mutations and instability affecting this locus are the molecular basis for the majority of grape colour sports.

White grapes are thought to have originated from a black-fruit ancestor via two independent mutations to the closely linked *VvMybA1* and *VvMybA2* genes. A Gret1 retrotransposon insertion into the promoter of *VvMybA1* and a small indel causing a frameshift mutation in *VvMybA2* inactivate both genes^82,83^. It is unclear exactly when the heterozygous red parent self-pollinated and gave rise to homozygous white progeny, but there is evidence that ancient Egyptians were making both red and white wine by 1332 BC^84^. Many of the white grape cultivars tested are homozygous for these same two mutations suggesting that most have a common origin^82,83, 85–87^. Loss of the Gret1 transposon in some sports restores *VvMybA1* function and gives rise to coloured revertants^82,86,88^. Other loss-of-pigment sports have been shown to arise from insertion of other types of TEs near *VvMyb*A^26^, short genomic insertions into the promoter or introns of *VvMyb*A^89^, or large-scale genomic replacements and rearrangements near *VvMyb*A^32,90^.

**Fig. 2:**
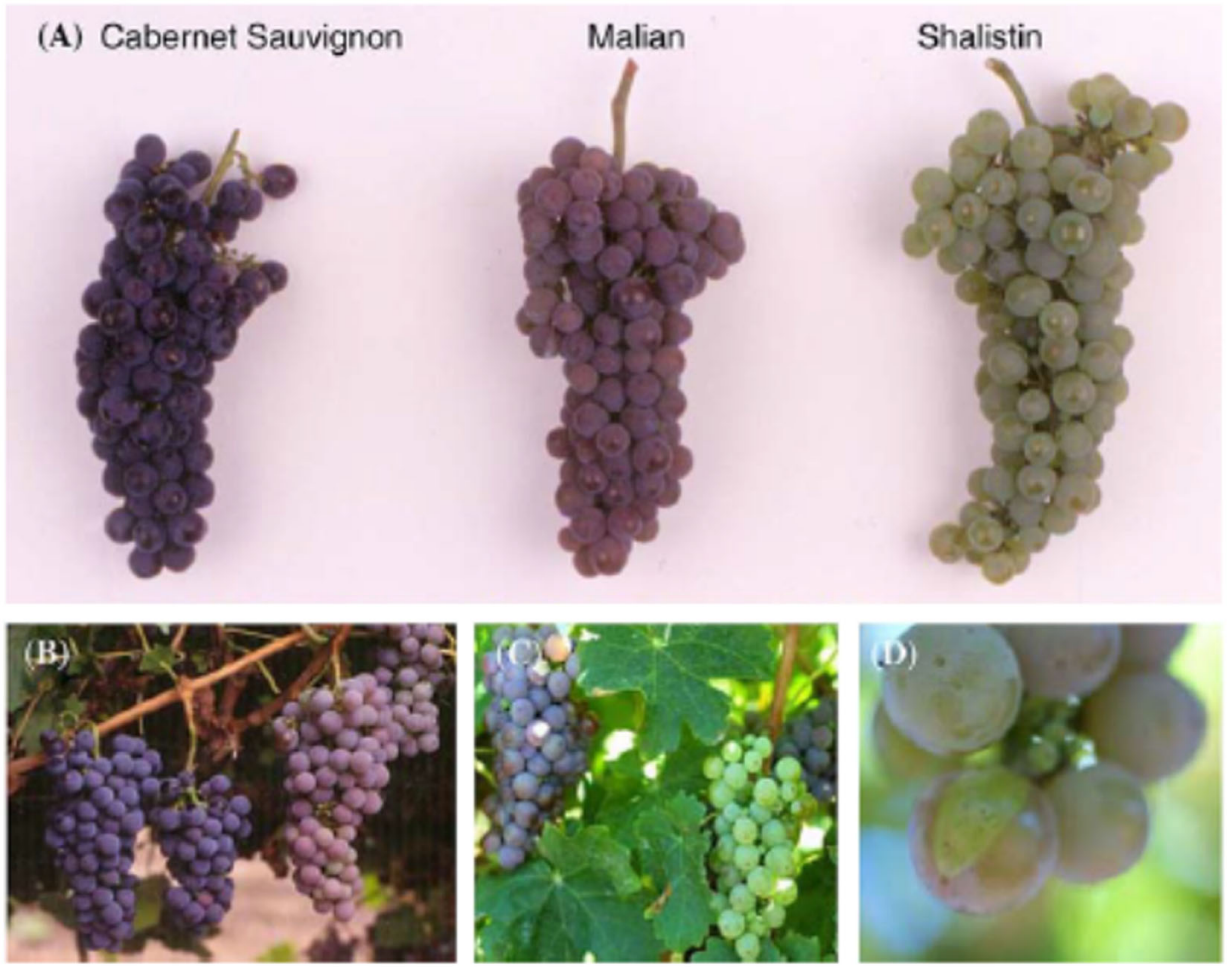
Photographs of coloured grape sports. **a** Bunches of Cabernet Sauvignon, Malian, and Shalistin. **b** A bunch bearing bronze berries (wild-type L1, mutant L2) on the original Cabernet Sauvignon plant. **c** A Malian vine with a white bunch (mutant L1 and L2). **d** A white sector on a Malian berry. *Source*: Walker et al.^91^

Dark skinned cultivars occasionally produce a bronze or pale coloured sport that eventually gives rise to white berries. Walker and co-workers demonstrated the molecular basis of two such examples^91^. Cabernet Sauvignon produces dark red berries, but is heterozygous for the mutant *VvMyb*A genes described above. A new mutation causing a large deletion of the functional *VvMyb*A genes occurred in the L2 of Malian, abolishing anthocyanin production in this layer and giving the berries a bronze colour. Malian is unstable and occasionally produces white grapes or white sectors following an invasion of mutant L2 cells into the L1 (Fig. 2). Pinot noir is another unstable red cultivar that gives rise to pale sports such as Pinot gris. The authors hypothesized that a separate deletion of the *VVMyb*A locus occurred in the L2, and eventually invaded the L1 to generate Pinot blanc clones, a stable white cultivar.

One of the most conspicuous fruit sports is the blood orange (*Citrus sinensis* L.), which requires exposure to cold to develop dark red fruit. The blood orange was first documented in Italy in 1646^92^ and was propagated clonally and sexually for centuries. A second blood orange sport arose in China in the late 1800s or earlier^93^. Both sports are caused by insertion of a retroelement near *Ruby*, a *Myb* gene that regulates anthocyanin production in fruit^94^. Cold induces retroelement transcription which activates transcription of Ruby. Many of the blood oranges found throughout Europe are derived from recombination between the LTRs that were maintained as periclinal chimeras^94^.

Epigenetic changes to *Myb* loci can also produce sports with altered colouration. “Ralls” and “Zaosu Red” are red fruit sports in apple (*Malus* ×  *domestica* Bork.) and a hybrid of Asian and European pear (*Pyrus pyrifolia* and *P. communis*), respectively. Both are associated with decreased methylation of promoter regions of orthologous genes *MdMYB1* and *PyMYB10*^95,96^. Conversely, “Blondee”, a yellow sport of a red parent, is associated with demethylation of the *MdMYB10* promoter^97^. Some apple cultivars can produce fruit with both solid and striped pigmentation. The regions of red stripes are associated with increased expression of *MdMYB10* and decreased methylation in the *MdMYB10* promoter^98^.

Yellow flesh peaches (*Prunus persica*) arose from at least three independent loss-of-function mutations to the *carotenoid cleavage dioxygenase4* (*PpCCD4*) gene^99,100^. These lesions are caused by an SNP introducing a premature stop, a dinucleotide (CT) insertion in a microsatellite region creating a frameshift mutation causing a premature stop, or the insertion of a retrotransposon into an intron. Genotyping yellow and white cultivars and somatic revertants demonstrated that yellow flesh peaches have two non-functional *PpCCD4* alleles, whereas the white progenitors and revertants have at least one functional *PpCCD4* allele, which is sufficient to block carotenoid accumulation in the flesh. Two white bud sport mutants, revertants from yellow cultivars, were used to validate the causal relationship between allele and phenotype. Both white sports have a yellow suture (an L1-derived tissue), indicating that these are periclinal chimeras comprised of a *PpCCD4*-deficient L1 and a revertant L2 that has restored one functional *PpCCD4* allele^99,100^. In one of the sports, the insertion of another CT in the mutated microsatellite region restored the reading frame and removed the stop codon^99^. In the other sport, deletion of the retrotransposon led to restoration of gene function^100^.

Pink or red sports in orange^101,102^, pummelo^103^ (*Citrus grandis* Osbeck.), and grapefruit^104^ (*Citrus paradisi* Macf.) have greatly increased accumulation of lycopene in the fruit. The genetic lesions causing the increase in lycopene accumulation have not been identified; however, one report showed that a cluster of six candidate genes exhibited gene dosage variation and decreased transcription between the sport Hong Anliu and its parent^105^. A yellow citrus sport Pinalate has yellow fruit with high levels of carotenes and decreased ABA content^106^. Three citrus sports with brown skin have been reported to be caused by defective chlorophyll degradation^107^, altered carotenoid accumulation^108^, and defective synthesis or accumulation of β-citraurin^108^.

## Altered fruit size or shape

After fruit colour, the most obvious type of sport mutant is one that has a different fruit size or shape. Final fruit size results from cell division and enlargement, and many fruit size sports are clearly affected in one of these processes. Increased DNA content caused by endoreduplication or chimeric polyploidization often results in larger cells and increased lateral organ and/or fruit size^109,110^. Interestingly, giant fruit sports in apple^111^ and pear^112^ both showed fruit-specific increases to DNA content and cell size, suggesting that fruit cell size is under separate regulation to other parts of the plant. Another giant pear sport had no change in ploidy, but showed increased expression of an actin-related protein that is involved with regulating cell proliferation in Arabidiopsis^113,114^.

“Totsutanenashi” (TTN) is a small fruit sport in Japanese persimmon (*Diospyros kaki* Thumb.) that also increases sugar content in fruit and causes a more compact tree architecture^115^. Application of exogenous cytokinin restores normal fruit size^116^, suggesting that the mutant gene involves cytokinin biosynthesis or signal transduction. The progenitor of TTN is non-aploid (2*n* = 9×) and has produced many sports, raising the possibility that recent increases in ploidy have led to increased transposon movement and/or chromosomal rearrangements.

In grape, the *fleshless berry* (*flb*) mutation disrupts cell division and differentiation in the mesocarp of the fruit, resulting in a 10-fold reduction in berry weight^117^. Observations that the *flb* phenotype was unstable and that some progeny obtained from sexual propagation showed a new phenotype that failed to set fruit led to the discovery that the original *flb* mutant is an L2 chimera^118^. The *flb* mutation is caused by the insertion of a TE into the promoter of *VvPI*, resulting in ectopic expression of the grape homologue of Arabidopsis *PISTILATA*^119,120^. Analysis of plants that carry the mutation in the L1, L2, or in both layers demonstrate the differential effects of ectopic *VvPI* expression in each layer. *VvPI* expression in the L2 blocks flesh development by preventing mesocarp cells from differentiating, while *VvPI* expression in the L1 and L2 disrupts carpel development at fruit set^119^. This is an excellent example of how the study of a sport mutant has provided new insights into the layer-specific role of PI in floral and fleshy fruit development. Interestingly, both the TTN and *flb* L2 sports have increased sugar content and decreased phenolic compounds, which suggest a potential connection between fruit size and biochemical properties^115,117^.

Nectarines were first identified in China over 2000 years ago^121^. Genotypic analysis indicates that nectarines were introduced or arose in Europe multiple times, probably as bud sports^122^. Genomic data from five peach/nectarine accessions indicated that the insertion of a LTR retroelement in the coding sequence of *PpeMYB25* is the likely cause of the hairless nectarine phenotype^123^. In Arabidopsis, loss-of-function mutations to the closely related MYB gene, *GLABRA1*, result in hairless leaves^124^. Nectarine sports of peach are frequently observed in orchards and some have been commercialized.

**Fig. 3:**
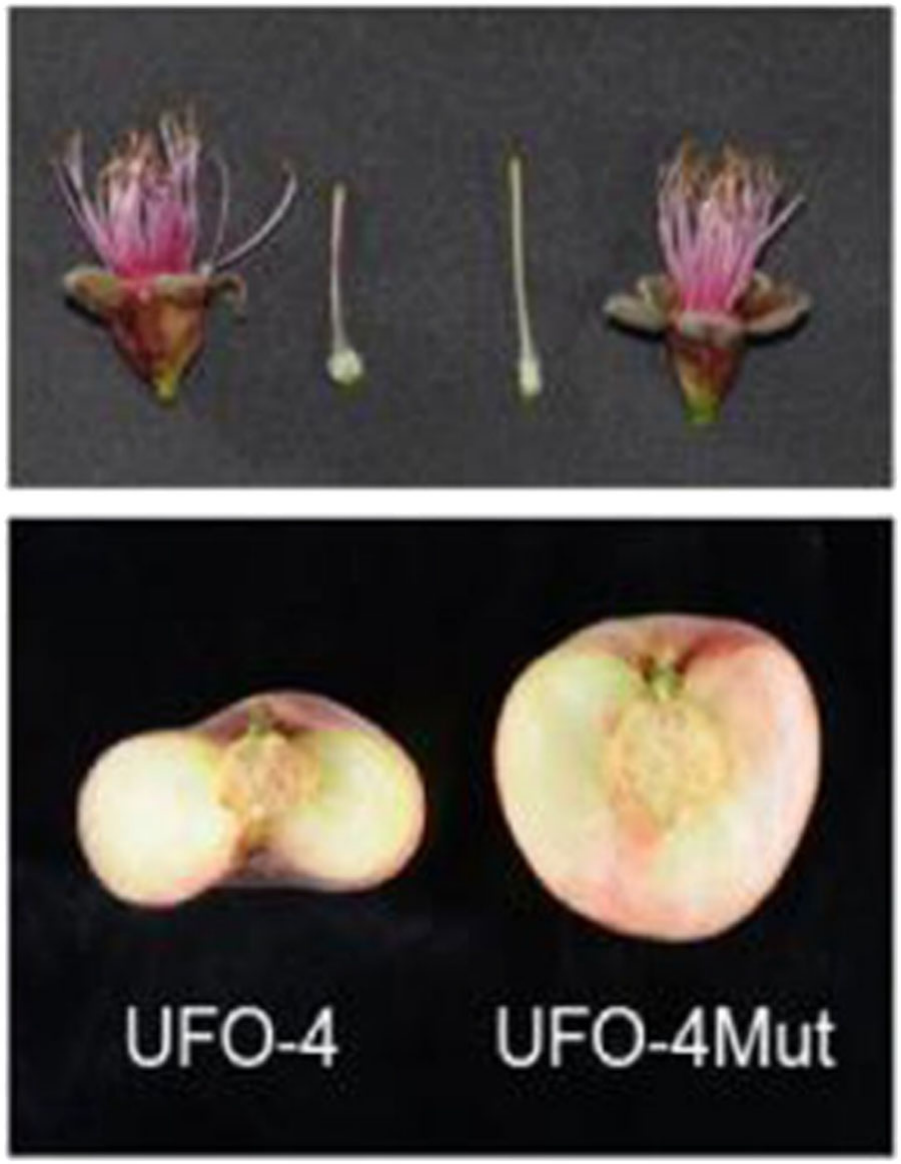
Image of the pistils and fruit from flat peach variety (UFO-4) and its round somatic mutant (UFO-4Mut). *Source*: López-Girona et al.^125^

Flat fruit shape in peach is caused by a single semi-dominant locus (S), which may itself be a bud sport mutant that originated in China. Individuals heterozygous for S have flat fruit, while those homozygous for S show early abortion of the fruit^125^. Analysis of the flat fruit indicates they have fewer cells in the vertical axis due to earlier cessation of cell division relative to round fruit^126^. The flat fruit phenotype has been associated with a 10 kb deletion that removes the first 693 bp of a leucine-rich receptor-like kinase (LRR-RLK) gene (*PRUPE*.6G281100/*ppa025511m*), orthologous to the Arabidopsis *BRASSINOSTEROID INSENSITIVE1 ASSOCIATED KINASE* (*BAK1*) group^125^. *BAK1*-like genes regulate cell division and meristem size in Arabidopsis^127,128^, rice^129, 130^, and maize^131^. A bud sport mutant reverting from flat to round (Fig. 3) has been shown to carry a new mutation to *PRUPE*.6G281100 in the L2 layer, providing strong support that this gene is involved with the flat fruit phenotype. Guo et al.^126^ identified some flat peach accessions with two functional *PRUPE*.6G281100 alleles, suggesting that a second gene may also contribute to this phenotype. A polymorphism in *PpCAD1/ppa003772m*, annotated as *CONSTITUTIVELY ACTIVATED CELL DEATH GENE1*, was highly associated with the flat peach phenotype in a genome-wide association study^132^. However, the SNP is not predicted to alter gene function and the gene is not differentially expressed between flat and round fruit until the mature fruit stage.

## Changes to plant architecture

**Fig. 4:**
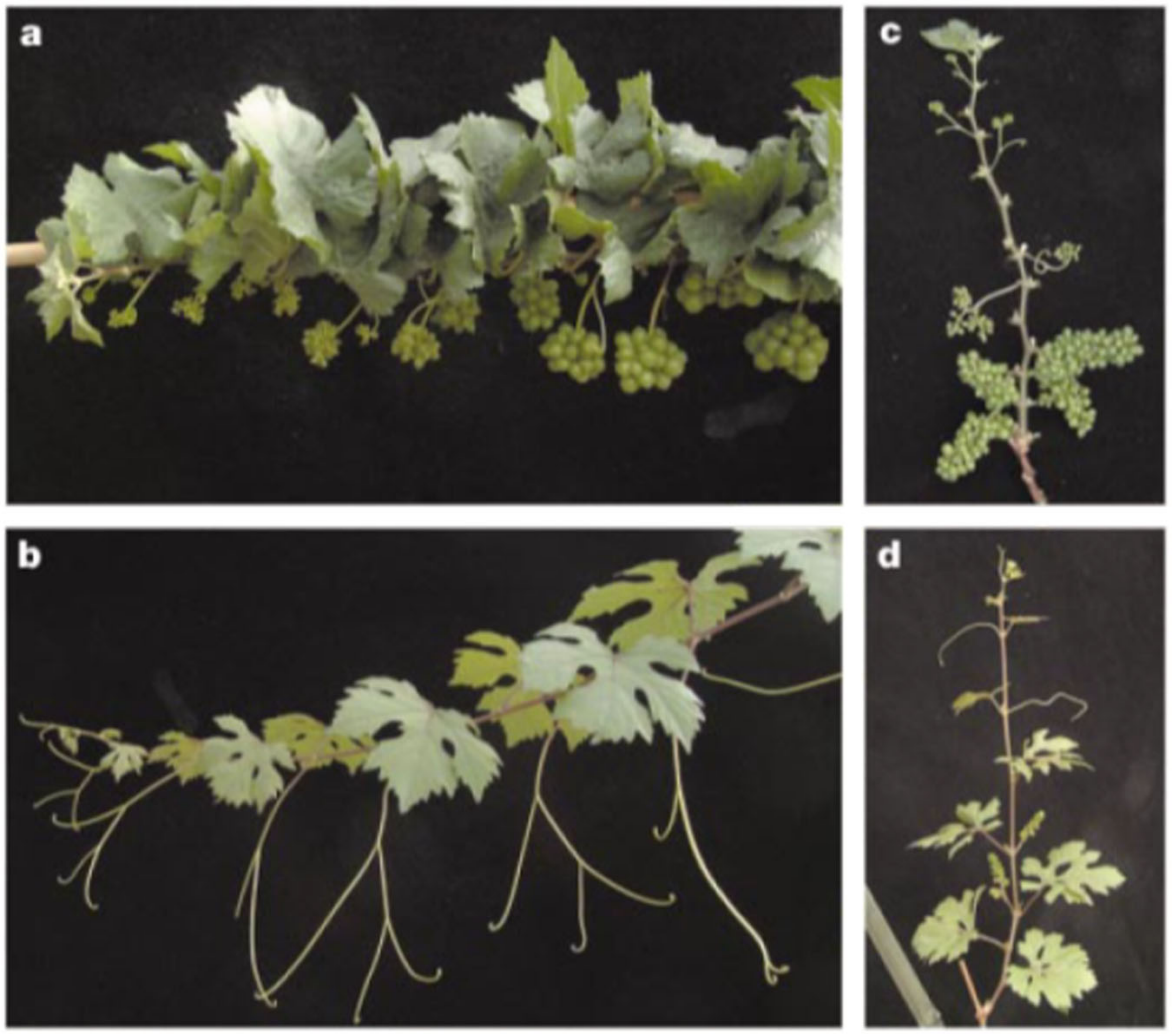
Pinot Meunier is a bud sport chimera with a dominant mutation to the *VvGAI* gene in the L1 layer. **a**, **c** A grapevine derived from the L1 of Pinot Meunier carrying the mutant *VvGAI* gene in both L1 and L2 layers. Note the conversion of tendrils to inflorescences and the shortened internodes. **b**, **d** A plant derived from the non-mutant L2 of Pinot Meunier

On rare occasions, a sport arises that drastically alters plant architecture. Pinot Meunier is a sport of Pinot noir that is characterized by having leaves and stems that are densely covered with trichomes and a conversion of tendrils into inflorescences^133^. Occasionally, leaf sectors lacking the hairy phenotype appear on Pinot Meunier suggesting that it is a periclinal chimera with a mutant L1^2,133^. Indeed, plants regenerated from L1 or L2 layers indicate L1-derived plants are hairy, while L2-derived plants are hairless^2^. More striking is the dwarfed phenotype of L1-derived plants, caused by very short internodes (Fig. 4a, c). This dwarfed phenotype was not rescued by application of gibberellins (GAs), indicating that it is not a GA biosynthesis mutant^134^. The mutation is caused by a non-synonymous SNP in the highly conserved DELLA domain of VvGAI, which encodes a member of key GA-responsive proteins^134^. The conversion of tendrils to inflorescences supports the idea that the grape tendril is a modified inflorescence normally inhibited from floral development by GA. This is another excellent example of sport mutants providing new information about the layer-specific effects of a mutation.

**Fig. 5:**
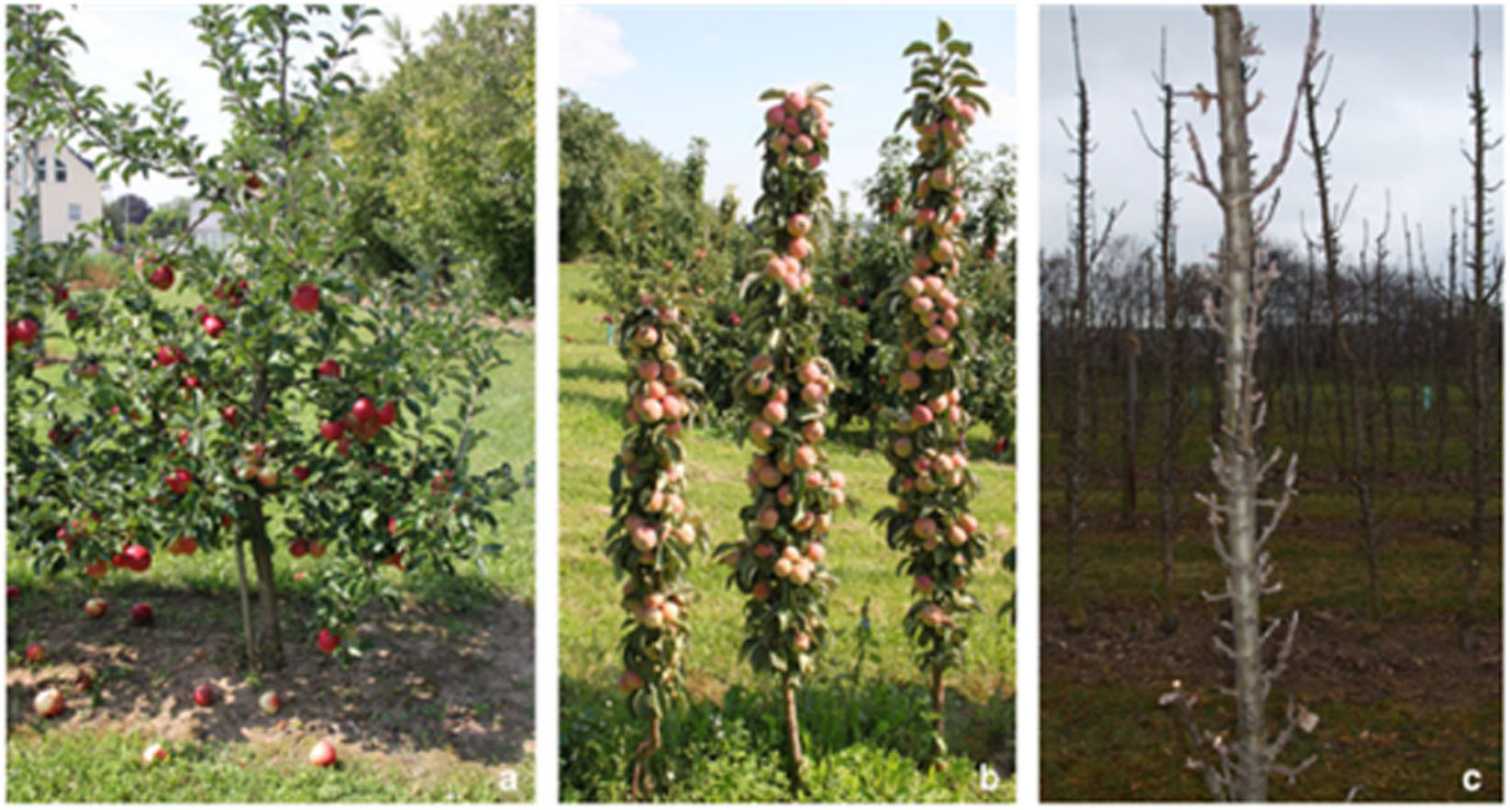
Comparison of the plant architecture of standard and columnar type apple trees. **a** Apple trees with a standard growth habit have long lateral branches and a wide branching angle relative to the primary shoot. **b**–**c** Columnar trees have a compact growth habit and produce mostly short fruit spurs with a narrow branch angle. *Source*: Peterson and Krost^155^

In the early 1960s, apple grower Anthony Wijcik noticed an abnormal shoot on a “McIntosh” tree in his orchard^135^. The “Wijcik” sport has a “columnar” growth habit with a thick, stunted primary axis, very short internodes, and short lateral spurs rather than lateral branches (Fig. 5). This phenotype has generated considerable commercial interest since columnar trees could be planted at high density, require less pruning than standard types, and potentially enable mechanical harvesting. The columnar phenotype segregates as a single dominant allele^136^ that is associated with the integration of a retrotransposon into the genome^36,137^. Although there is some discrepancy in the size of the insertion, it is clear that the insertion does not disrupt any coding sequence, but does alter expression of nearby genes. Expression analysis of genes within 25 Kb of the insertion demonstrated that a gene encoding a putative 2OG-Fe (II) oxygenase was upregulated 14-fold over “McIntosh” in young axillary buds. Overexpression of this gene (*MdCo31*) in Arabidopsis resulted in very short inflorescences due to reduced internode lengths^137^. Members of this gene family are involved with the biosynthesis of ethylene, flavonoids, gibberellins, and defence against downy mildew. Several studies have used RNAseq to identify differentially expressed genes (DEGs) between columnar and standard trees^36,138,139^. Many of the DEG from shoot meristem tissue are involved with hormone metabolism and signalling^140^. Genes involved with lignin and terpene biosynthesis, and pathogen/pest attack response were highly upregulated in “Wijcik” leaves^36^. Two other genes near the insertion are upregulated in “Wijcik”. One encodes a helix-loop-helix transcription factor and the other *Downy Mildew Resistant 6* (*DMR6*), a regulator of defence genes.

## Somatic mutants of non-perennial species

Some annual crop plants are clonally propagated for a variety of reasons. Clonal propagation preserves the genotype and phenotype of the parent plant, which could be lost in sexual propagation. Prevention of sexual reproduction helps avoid inbreeding depression which is common in outcrossing species. In varieties selected for seedlessness, such as banana, sexual propagation may not be possible.

A large number of potato (*Solanum tuberosum*) cultivars are bud sports that alter the pigmentation of the tuber including the leading French fry processing cultivar “Russet Burbank“^141–145^. Many coloured potato sports are periclinal chimeras that show a range of pigmentation from white to purple^143–145^. Careful analysis of colouration patterns in these sports and their sexual offspring revealed that the L1 gives rise to most of the tuber skin except for a small patch below each eye, which is L2 derived^143^. The “Kostroma mutant” does not affect the tuber, but shows pronounced dissection of leaf blade and flower corolla^146^. Spontaneous mutations giving rise to coloured tubers and sectorial chimeras have also been developed into popular cultivars of sweetpotato (*Ipomoea batatas*)^147^.

Cassava (*Manihot esculenta* Crantz) is an important staple crop in Sub-Saharan Africa, the tropics, sub-tropics, and South Pacific islands. Cassava sports with altered leaf morphology and increased vigour are associated with spontaneous changes in ploidy, probably caused by the fertilization of unreduced gametes^148–150^. Many of the crops grown in Oceania originated from a very limited number of introduced genotypes and have been mostly or exclusively vegetatively propagated. Somaclonal variation has been a valuable source of phenotypic diversity for farmers growing taro (*Colocasia esculenta* Schott) and yam (*Dioscorea alata* L.)^151^, bananas and plantains (*Musa* spp.)^152,153^, and kava (*Piper methysticum* Forst f.)^154^.

## Conclusions

Bud sport mutants introduce new genetic variability, which is crucial for species that lack variability or cannot be sexually reproduced. Somatic mutations can also provide valuable new characteristics while retaining the desirable qualities of the parent plant, which is why many popular cultivars have originated from sports. In some cases, the analysis of sports has revealed new information about layer-specific roles of some genes or has provided valuable insight into gene function during specific developmental processes. As sequencing technologies improve, sports also provide a means to examine mechanisms that drive genomic evolution.
